# Supplementary material for: Solvation, Cancer Cell Photoinactivation and the Interaction of Chlorin Photosensitizers with a Potential Passive Carrier Non-Ionic Surfactant Tween 80
Source: Int J Mol Sci. 2022 May 10;23(10):5294. doi: 10.3390/ijms23105294 (PMC9140634; doi:10.3390/ijms23105294)
Supplement: Supplementary file 1 [file ijms-23-05294-s001.zip › ijms-1707113-supplementary.pdf]

Supplementary Material File for

Solvation, cancer cell photoinactivation and the interaction of chlorin photosensitizers with a potential passive carrier non-ionic surfactant Tween 80

by Andrey V. Kustov *et al*.

1. Synthesis of chlorin photosensitizers (comps. 1-4, see Table 1 in the main text and the structures below)

The absorption and fluorescence spectra of comps. **2**, **3** are presented in Figure S0. The spectra of comps. **1, 4** are given elsewhere [S1, S2].

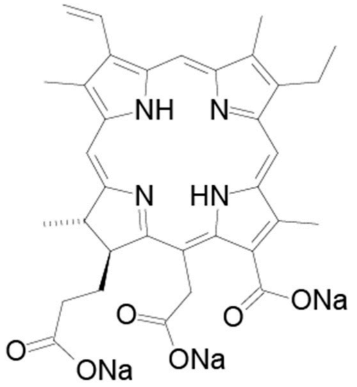	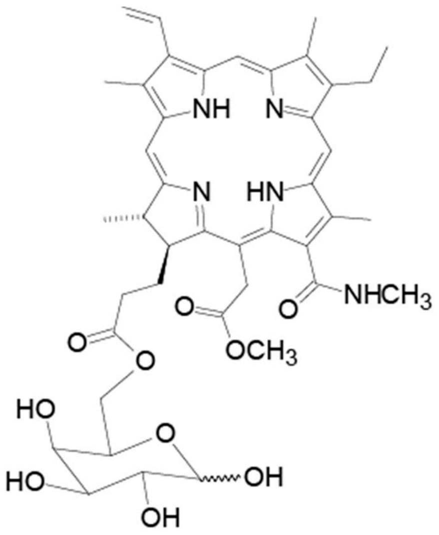
Chlorin e_6 trisodium salt (comp. **1**)	Chlorin e_6 13(1)-N-methylamide-15(2)-methyl ester-17(3)-O-6'-galactopyranosyl ester (comp. **2**)
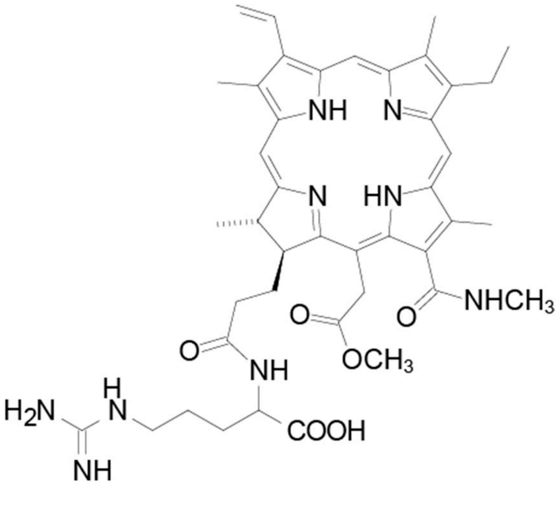	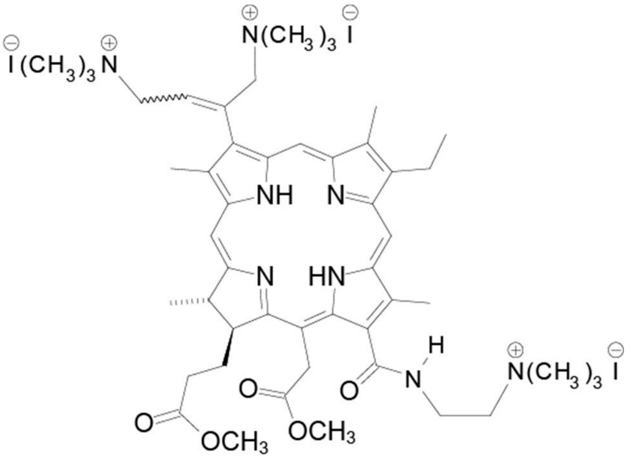
Chlorin e_6 13(1)-N-methylamide-15(2)-methyl ester-17(3)-[N-1′-(1′-carboxy-4′-guanidylbutyl) amide] (comp. **3**)	Chlorin e_6 3(1),3(2)-*bis*-(N,N,N-trimethylamino-methyl iodide)-13(1)-N'-(2-N'',N'',N''-trimethyl ammonioethyl iodide) amide 15(2),17(3)-dimethyl ester (comp. **4**)

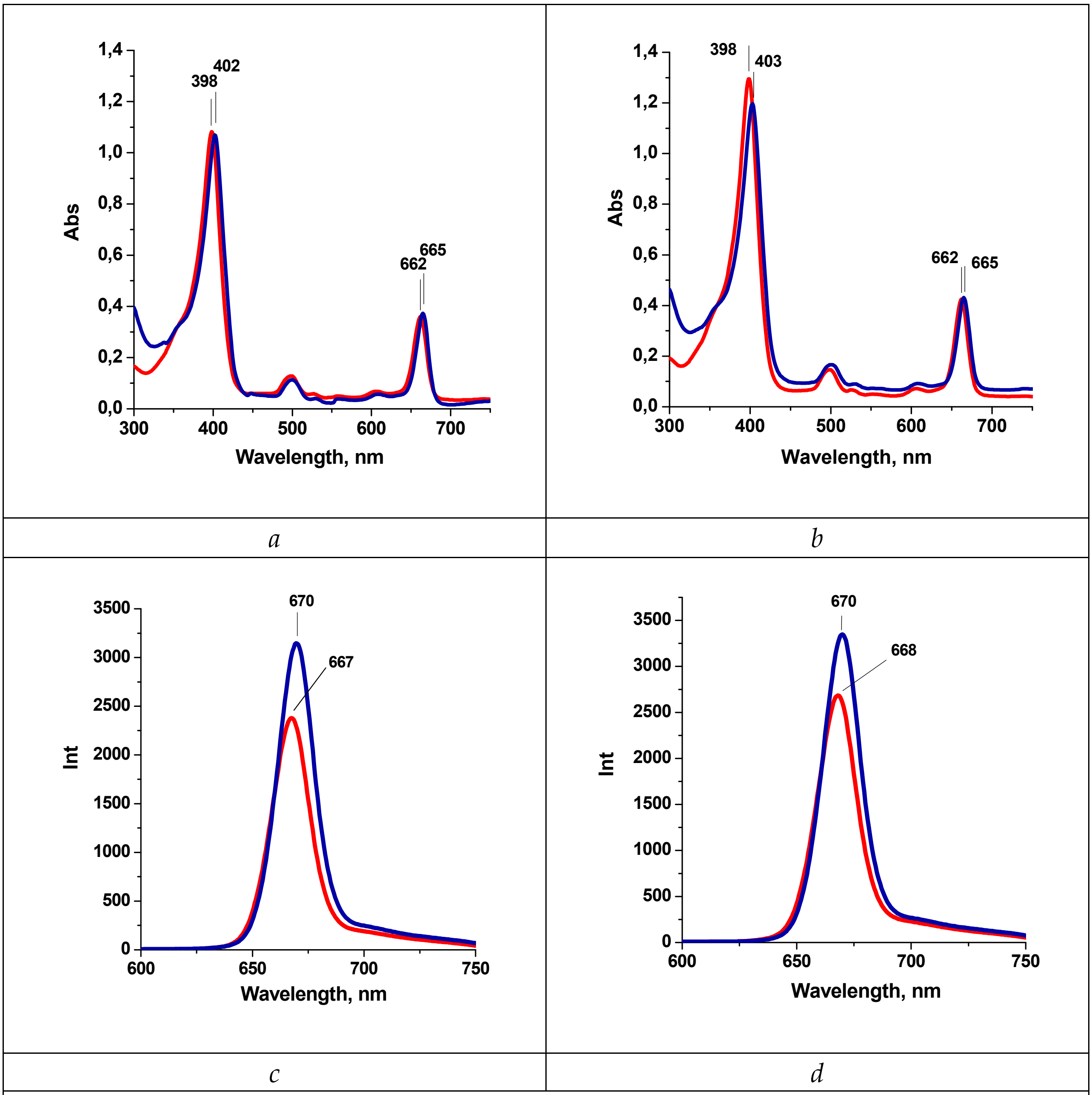

Figure S0: Absorption (*a, b*; ~ $1 \cdot 10^{-5}$ mol/kg) and fluorescence (*c, d*; ~ $1 \cdot 10^{-6}$ mol/kg) spectra of the PSs studied: (*a, c*) – comp. **2**; (*b, d*) – comp. **3**. The blue lines show the results in 1% aqueous solution of Tween 80, while the red lines refer to EtOH.

1.1 Chlorin e_6 trisodium salt (comp. **1**, the so-called PS “Fotoran e_6”) was purchased from “RANFARMA” company (Russia) as a solid powder mixed with polyvinylpyrrolidone (PVP) and reprecipitated from an aqueous solution to obtain a pure solid form of comp. **1**. Purification of the PS from PVP was carried out as follows: 50 mg of Fotoran e_6 was dissolved in 20 ml of distilled water; then diluted hydrochloric acid was added dropwise to achieve pH~6.0. The precipitate of chlorin e_6 was centrifuged, separated from a liquid phase and washed several times with distilled water. The resulting dark-blue powder (18 mg) was dissolved in 20 ml of diluted aqueous solution of sodium hydroxide (20 ml, pH~8). The solvent was removed using a rotary evaporator under vacuum and then the solid was carefully dried. The yield of comp. **1** was 40 %. The NMR spectrum is presented in Figure S1.

^{1}H NMR (500 MHz, DMSO d_6, *J*, Hz), δ, ppm: 9.75 (s, 1H, H-10); 9.60 (s, 1H, H-5); 9.08 (s, 1H, H-20); 8.33 (m, 1H, H-3(1)); 6.43 (d, *J* = 16.0, 1H, H-3(2) (*trans*)); 6.13 (d, *J* = 10.5, 1H, H-3(2) (*cis*)); 5.66 (br. s, 2H, CH_2-15(1)); 4.54 (m, 2H, H-18, H-17); signals from 4 to 1 ppm seem to be covered by residual PVP; -2.06 (br.s, 1H, 21-NH); -2.62 (br.s, 1H, 23-NH).

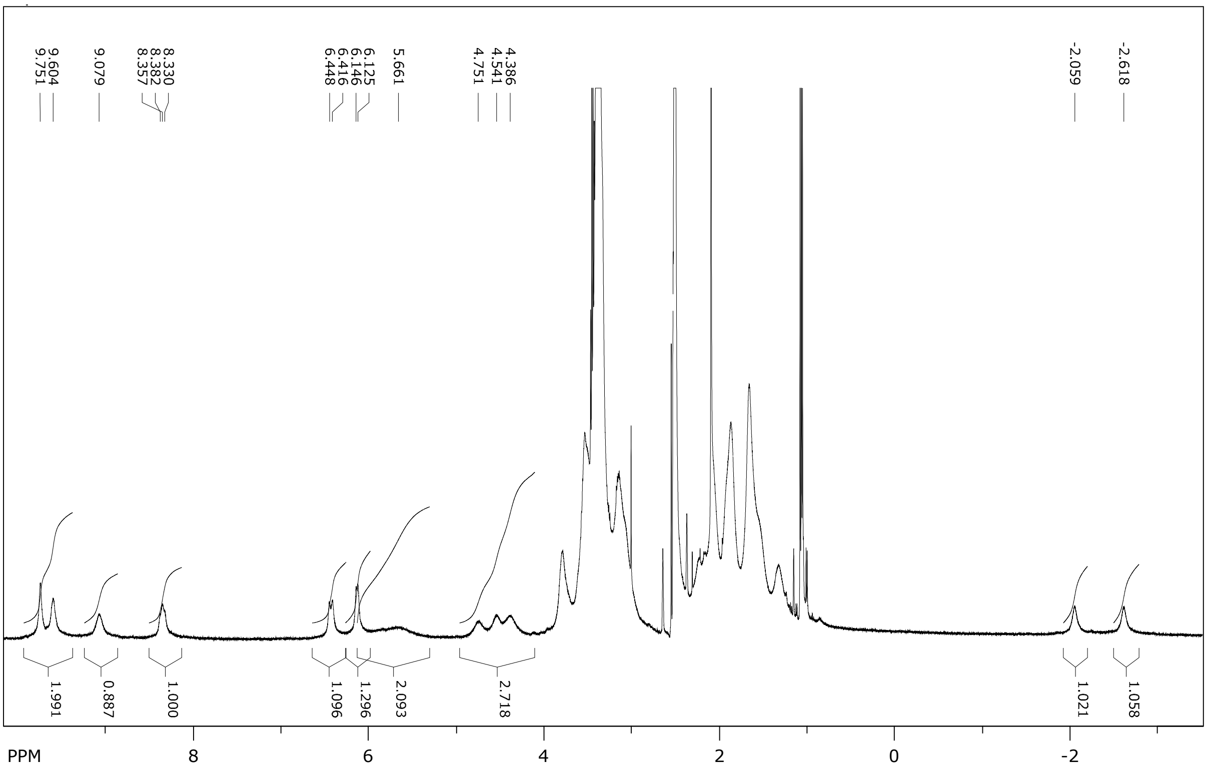

Figure S1: ^{1}H NMR spectrum of chlorin e_6 trisodium salt (comp. **1**) in DMSO d_6 (the sample contained PVP in the PS/PVP molar ratio of 100/1).

1.2 Chlorin e_6 13(1)-N-methylamide-15(2)-methyl ester-17(3)-O-6'-galactopyranosyl ester (comp. 2)

The amphiphilic conjugate of chlorin e_6 derivative with *D*-galactose (comp. **2**) was synthesized according to a recently published procedure [S3] by means of the interaction of activated 17(3)-carboxylic group of a chlorin PS with a protected *D*-galactose derivative, followed by hydrolysis of protecting groups (see Scheme S1). The MS and NMR spectra for comp. **2** are presented in Figures S2–S4.

Mass spectrum (ESI), m/z: 786.7 $[M+H]^+$.

^{1}H NMR (300 MHz, DMSO d_6, *J*, Hz), δ, ppm: 9.81 (s, 1H, H-10); 9.77 (s, 1H, H-5); 9.15 (s, 1H, H-20); 9.07 (m, 1H, NH-13(1) (amide)); 8.33 (dd, *J* = 11.6, 17.9, 1H, H-3(1)); 6.46 (d, *J* = 17.4, 1H, H-3(2) (trans)); 6.19 (d, *J* = 12.0, 1H, H-3(2) (cis)); 5.54 (d, *J* = 19.2, 1H), 5.37 (m, 1H) (CH_2-15(1)); 4.89 (d, *J* = 12.3, 1H, H-1[a]); 4.66 (m, 1H, H-18); 4.50 (d, *J* = 9.3, 1H, H-17); 4.65 (d, *J* = 7.2, 2H,), 4.19-3.89 (m, 4H) (H-2[a], H-3[a], H-4[a], H-5[a], CH_2-6[a]); 3.84 (q, *J* = 7.3, 2H, CH_2-8(1)); 3.71 (s, 3H, CH_3-15(3)); 3.54 (s, 3H, CH_3-12(1)); 3.51 (s, 3H, CH_3-2(1)); 3.33 (s, 3H, CH_3-7(1)); 3.67-3.42 (m, 4H, OH-1[a], OH-2[a], OH-3[a], OH-4[a]); 3.13 (d, *J* = 3.9, 3H, CH_3-13(2)); 2.80-2.55 (m, 2H), 2.26-2.04 (m, 2H) (CH_2-17(1), CH_2-17(2)); 1.77-1.59 (m, 6H, CH_3-18(1), CH_3-8(2)); -1.82 (br.s, 1H, 21-NH); -2.09 (br.s, 1H, 23-NH).

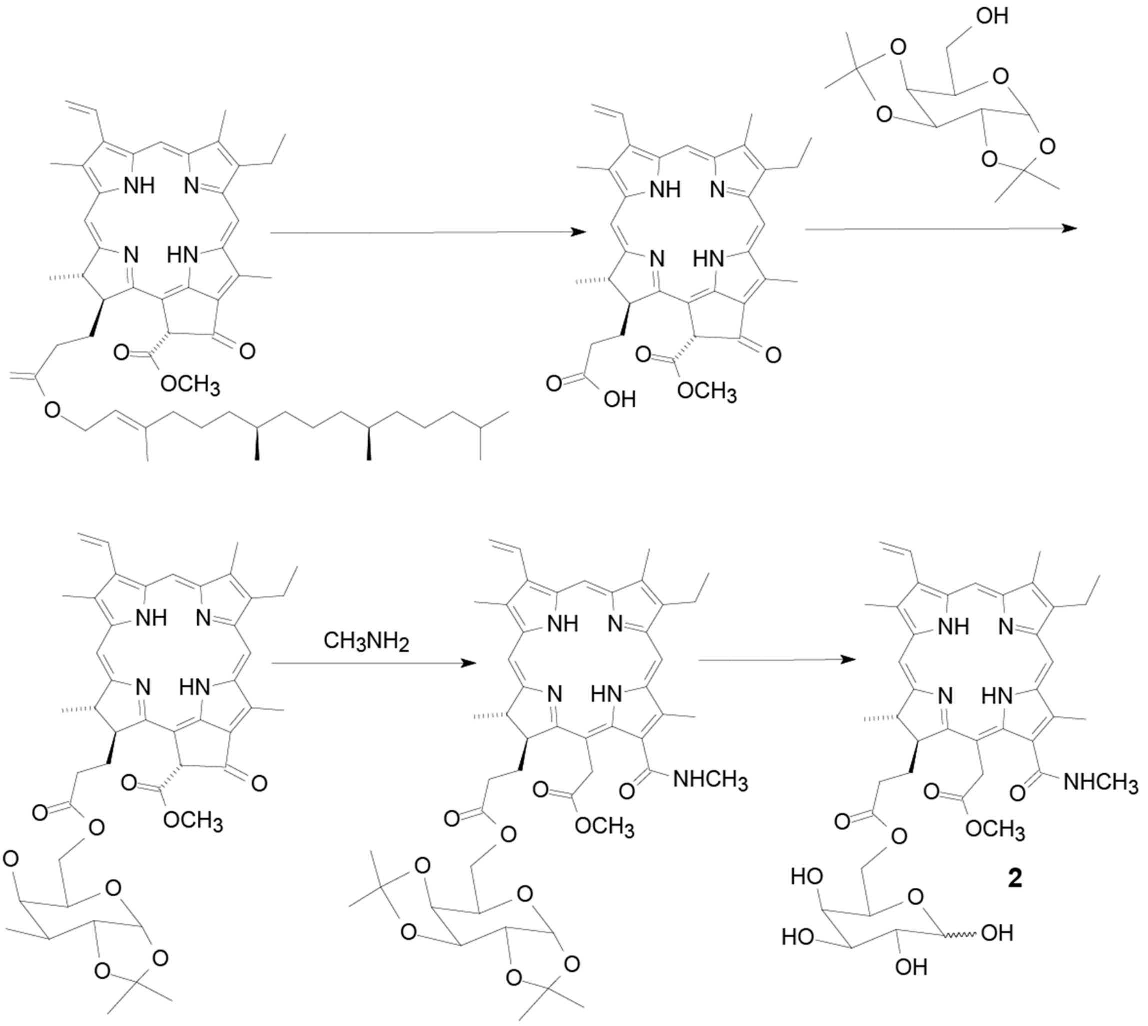

Scheme S1: Synthesis of chlorin e₆ 13(1)-N-methylamide-15(2)-methyl ester-17(3)-O-6'-galactopyranosyl ester (comp. **2**).

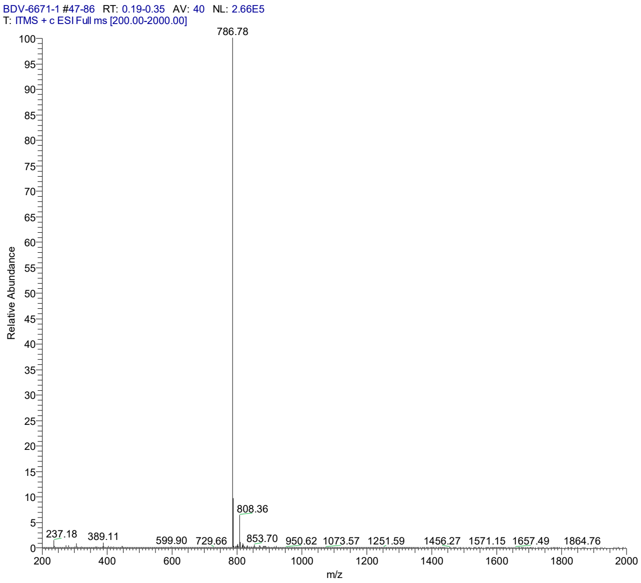

Figure S2: Mass spectrum (electrospray, ESI) of chlorin e_6 13(1)-N-methylamide-15(2)-methyl ester-17(3)-O-6'-galactopyranosyl ester (comp. **2**).

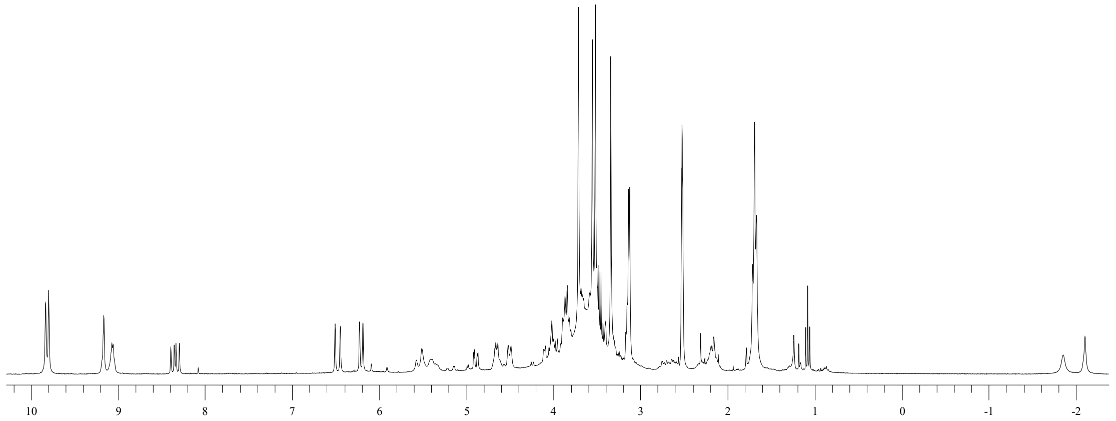

Figure S3: ^{1}H NMR spectrum of chlorin e$_6$ 13(1)-N-methylamide-15(2)-methyl ester-17(3)-O-6'-galactopyranosyl ester (comp. **2**) in DMSO d$_6$.

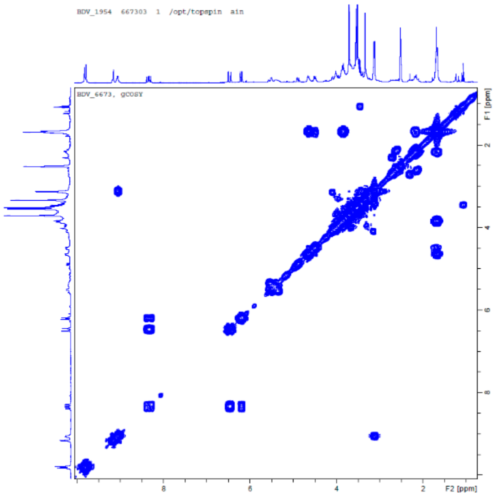

Figure S4: ^{1}H-^{1}H 2D NMR spectrum (COSY) of chlorin e$_6$ 13(1)-N-methylamide-15(2)-methyl ester-17(3)-O-6'-galactopyranosyl ester (comp. **2**) in DMSO d$_6$.

1.3 Chlorin e$_6$ 13(1)-N-methylamide-15(2)-methyl ester-17(3)-[N-1′-(1′-carboxy-4′-guanidylbutyl) amide] (comp. 3)

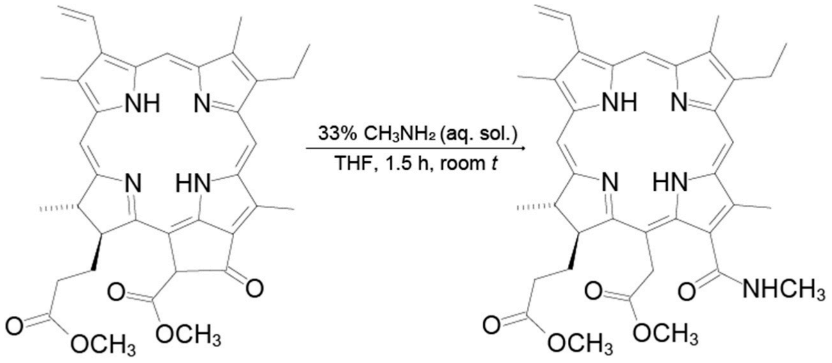

3a

Scheme S2: Synthesis of chlorin e$_6$ 13(1)-N-methylamide-15(2),17(3)-dimethyl ester (**3a**) from methylpheophorbide *a*.

Synthesis of the novel *L*-arginyl conjugate of chlorin e$_6$ (comp. **3**) was performed from methylpheophorbide *a* using a carboxylic group activation reaction through the formation of succinimide ester of PS according to Schemes S2-S5.

1.3.1. Chlorin e$_6$ 13(1)-N-methylamide-15(2),17(3)-dimethyl ester [S4].

The NMR spectra for comp. **3a** are presented in Figure S5.

Mass spectrum: m/z (MALDI) (%): M = 638.8776 (100) $[M]^+$; 660.9716 (10) $[M+Na]^+$.

^{1}H NMR (500 MHz, $CDCl_3$), δ, ppm: 9.74 (s, 1H, 10-H); 9.68 (s, 1H, 5-H); 8.85 (s, 1H, 20-H); 8.12 (dd, *J* = 11.5, 18.0 Hz, 1H, 3(1)-H); 6.43 (br. s., 1H, 13-NH); 6.38 (d, *J* = 18.0 Hz, 1H, 3(2)-H-trans); 6.18 (d, *J* = 11.5 Hz, 1H, 3(2)-H-cis); 5.56 (d, *J* = 19 Hz, 1H, 15(1)-CH); 5.28 (d, *J* = 19.0 Hz, 1H, 15(1)-CH); 4.50 (q, *J* = 7.0 Hz, 1H, 18-H); 4.38 (d, *J* = 9.5 Hz, 1H, 17-H); 3.86 (s, 3H, 15(3)-CH_3); 3.82 (q, *J* = 7.5 Hz, 2H, 8(1)-CH_2); 3.64 (s, 3H, 17(4)-CH_3); 3.58 (s, 3H, 12(1)-CH_3); 3.52 (s, 3H, 2(1)-CH_3); 3.35 (s, 3H, 7(1)-CH_3); 3.28 (d, *J* = 5.0 Hz, 3H, 13(2)-NCH_3); 2.56 (m, 1H), 2.18 (m, 2H), 1.78 (m, 1H): 17(1)-, 17(2)-CH_2; 1.74 (d, *J* = 7.25 Hz, 3H, 18(1)-CH_3); 1.75 (t, *J* = 7.5 Hz, 2H, 8(2)-CH_3); -1.82 (s, 1H, 21-NH); -1.65 (s, 1H, 23-NH).

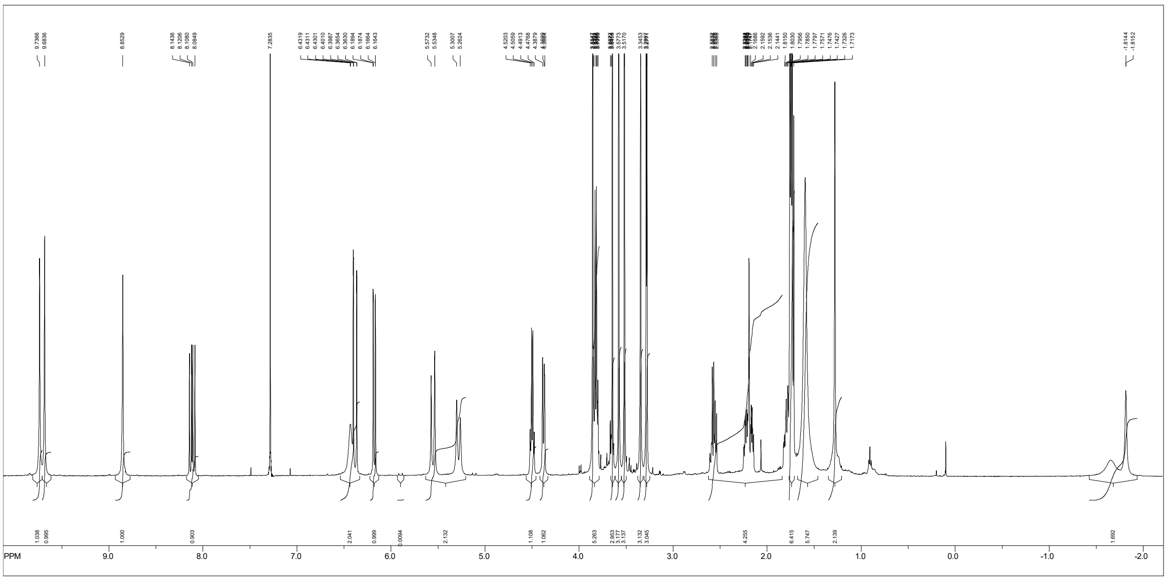

Figure S5: ^{1}H NMR spectrum of chlorin e$_6$ 13(1)-N-methylamide-15(2),17(3)-dimethyl ester (**3a**) in $CDCl_3$.

*1.3.2. Chlorin e$_6$ 17(3)-carboxy-13(1)-N-methylamide-15(2)-methyl ester (**3b**)*

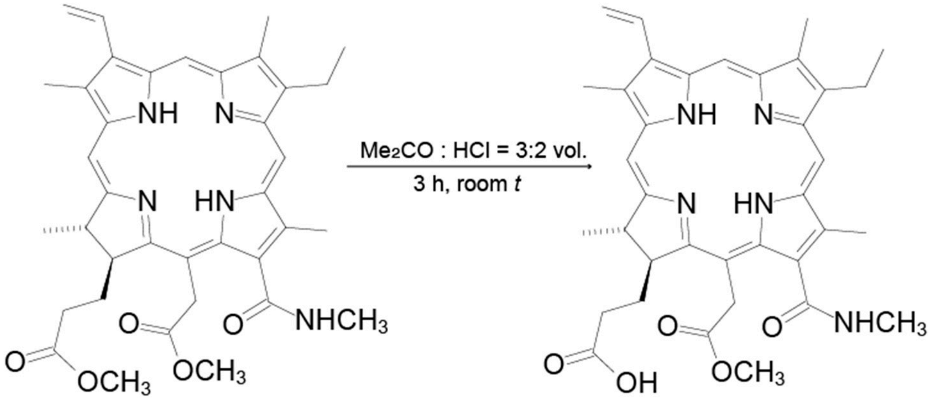

3a **3b**

Scheme S3: Synthesis of chlorin e$_6$ 17(3)-carboxy-13(1)-N-methylamide-15(2)-methyl ester (**3b**).

155 mg (0.243 mmol) of 13(1)-N-methylamide 15(2),17(3)-dimethyl ester of chlorin e_6 (comp. **3a**) was dissolved in 3 ml of acetone. 2 ml of concentrated hydrochloric acid was added and the reaction mixture was stirred for 3 hours at a room temperature. Then chloroform (5 ml) and water (10 ml) were added. The mixture was extracted with chloroform (3×10 ml) after that the extracts obtained were combined and additionally washed by water three times. The organic layer was separated and the solvent evaporated. Column chromatography of the crude product was carried out on silica gel (Fluka, 60 Å) using the dichloromethane-methanol mixtures as an appropriate eluent. The dichloromethane - methanol mixture (2 vol. %) bound to side products and removed them. Then, the main amount of the target product was washed out from the column with the dichloromethane - methanol mixture (10 vol. %). It was found to be acceptable to increase the concentration of methanol in dichloromethane up to 20 vol. % in order to collect additional quantities of the product. However, the further increase in the concentration of methanol lead to contamination of the product with more polar impurities. The product yield was 42 mg (28%).

The NMR spectra for comp. **3b** are presented in Figure S6.

Mass spectrum: m/z (MALDI) (%): M = 624.7900 (100) $[M]^+$
^{1}H NMR spectrum (500 MHz, $CDCl_3$), δ, ppm: 9.71 (s, 1H, 10-H); 9.65 (s, 1H, 5-H); 8.81 (s, 1H, 20-H); 8.11 (dd, *J* = 11.4, 17.9Hz, 1H, 3(1)-H); 6.40 (br. d, *J* = 4.3 Hz, 1H, 13-NH); 6.37 (d, *J* = 1.2, Hz, 1H, 3(2)-H-trans); 6.16 (d, *J* = 1.0 Hz, 1H, 3(2)-H-cis); 5.50 and 5.32 (both d, *J* = 19.5 Hz, 1H each, 15(1)-CH_2); 4.46 (q, *J* = 7.2 Hz, 1H, 18-H); 4.38 (d, *J* = 9.9 Hz, 1H, 17-H); 3.81 (s, 3H, 15(2)-$COOCH_3$); 3.57 (s, 3H, 12(1)-CH_3); 3.50 (s, 3H, 2(1)-CH_3); 3.33 (s, 3H, 7(1)-CH_3); 3.27 (d, *J* = 4.8 Hz, 3H, 13(2)-NCH_3); 2.09 – 2.62 (m, 4H, 17(1)-, 17(2)-CH_2); 1.73 (t, *J* = 7.8 Hz, 3H, 8(2)-CH_3); 1.72 (d, *J* = 7.8 Hz, 3H, 18(1)-CH_3); 0.87 (br. s, 1H , 21-NH), -1.80 (br. s, 1H, 23-NH).

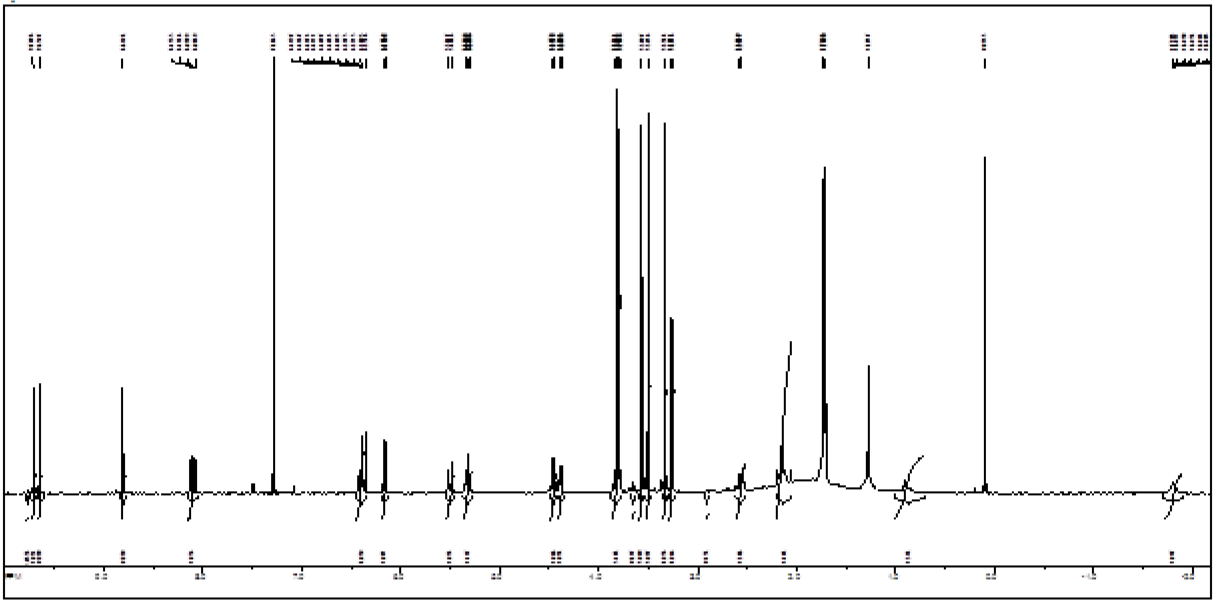

Figure S6: ^{1}H NMR spectrum of chlorin e_6 17(3)-carboxy-13(1)-N-methylamide-15(2)-methyl ester (**3b**) in $CDCl_3$.

*1.3.3. Chlorin e_6 13(1)-N-methylamide-15(2)-methyl ester-17(3)-succinimide ester (**3c**)*

41.9 mg (0.0670 mmol) of 17(3)-carboxy-13(1)-N-methylamide-15(2)-methyl ester (**3b**) of chlorin e_6, 9.4 mg (0.0817 mmol) of N-hydroxysuccinimide (NHS) and 34.6 mg (0.1680 mmol) dicyclohexylcarbodiimide (DCC) were dissolved in the mixture of 8 ml chloroform and 1 ml

tetrahydrofuran (THF). The reaction mixture was stirred for 24 h at a room temperature, then ~10 ml of chloroform was added and the mixture was washed with water, an aqueous solution of acetic acid (few drops) and water twice. The organic layer was separated and the solvent evaporated. The product contained some impurities (yield without additional purification was 73.3 mg or 100 %). The product was further used without purification.

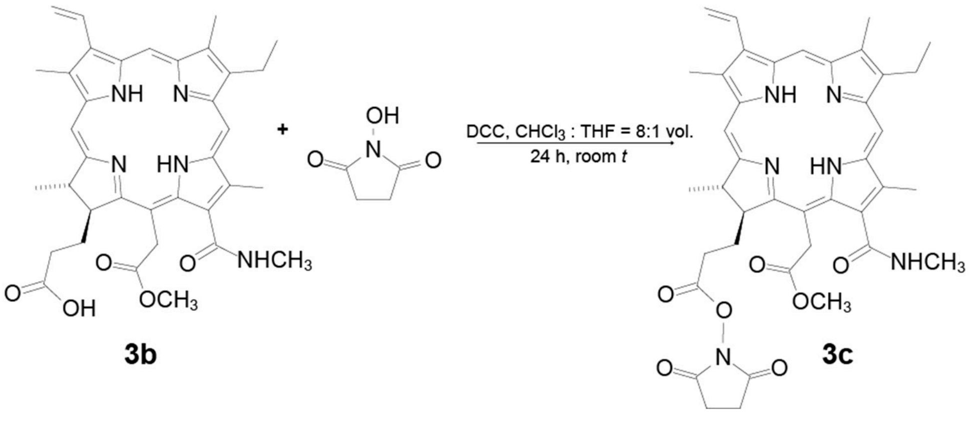

Scheme S4: Synthesis of chlorin e_6 13(1)-N-methylamide-15(2)-methyl ester-17(3)-N-succinimide ester (**3c**).

The NMR spectra for comp. **3c** are shown in Figure S7.

Mass spectrum: m/z (MALDI) (%): M = 722.4 (100) $[M]^+$

^{1}H NMR spectrum (500 MHz, $CDCl_3$), δ, ppm: 9.80 (s, 2H, 10-H, 5-H) ; 8.95 (s, 1H, 20-H); 8.12 (dd, *J* = 10.7, 18.5 Hz, 1H, 3(1)-H); 6.40 (d, *J* = 17.5 Hz, 1H, 3(2)-H-trans); 6.23 (br. d, *J* = 11.7 Hz, 1H, 3(2)-H-cis); 5.55 (d, *J* = 16.6 Hz, 1H, 15(1)-CH); 5.27 (d, *J* = 18.6 Hz, 1H, 15(1)-CH); 4.58 (s, 1H, 18-H); 4.06 (s, 1H, 17-H); 3.85 (s, 3H, 15(3)-CH_3); 3.60 (s, 3H, 12(1)-CH_3); 3.50 (s, 3H, 2(1)-CH_3); 3.38 (s, 3H, 7(1)-CH_3); 3.28 (m, 3H, 13(2)-NCH_3); 2.86 (s, 4H, NHS fragment); 1.95 (m, 1 H); 1.84 (m, 3 H) - 17(1)-, 17(2)-CH_2; 1.72 (tr, 3H, 8(2)-CH_3); 0.90 (m, 1H, 21-NH); -1.81 (s, 1H, 23-NH).

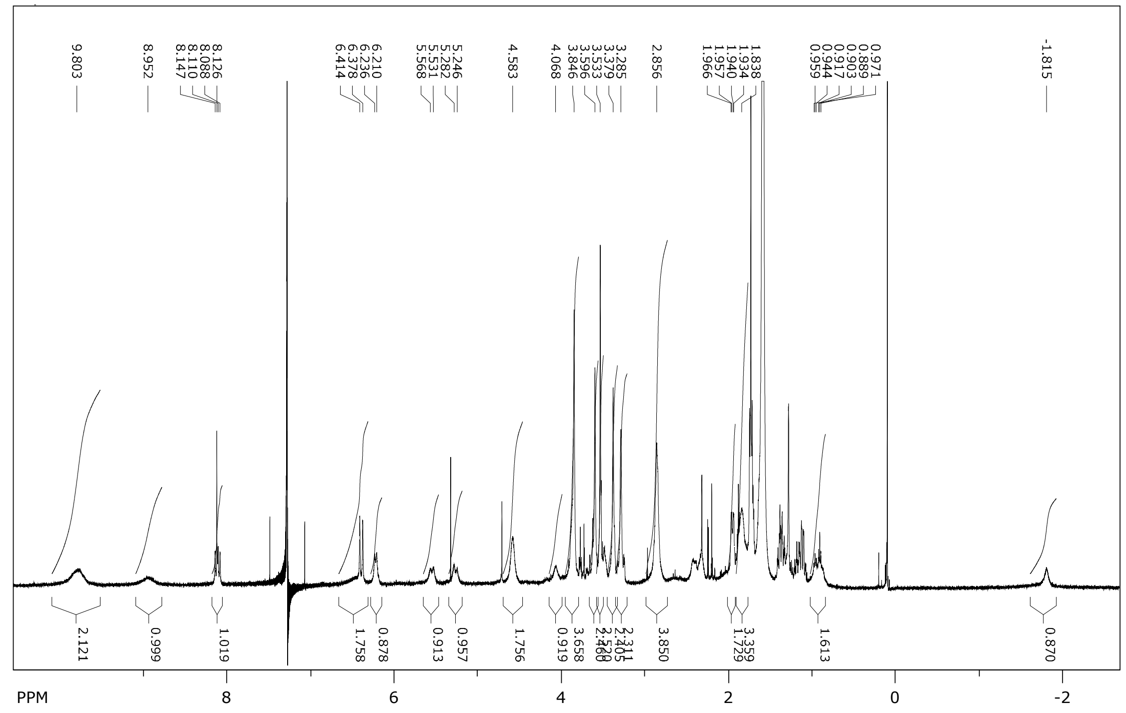

Figure S7: ^{1}H NMR spectrum of chlorin e_6 13(1)-N-methylamide-15(2)-methyl ester-17(3)-N-succinimide ester (**3c**) in $CDCl_3$.

*1.3.4 Chlorin e₆ 13(1)-N-methylamide-15(2)-methyl ester-17(3)-[N-1′-(1′-carboxy-4′-guanidylbutyl) amide] (comp. **3**).*

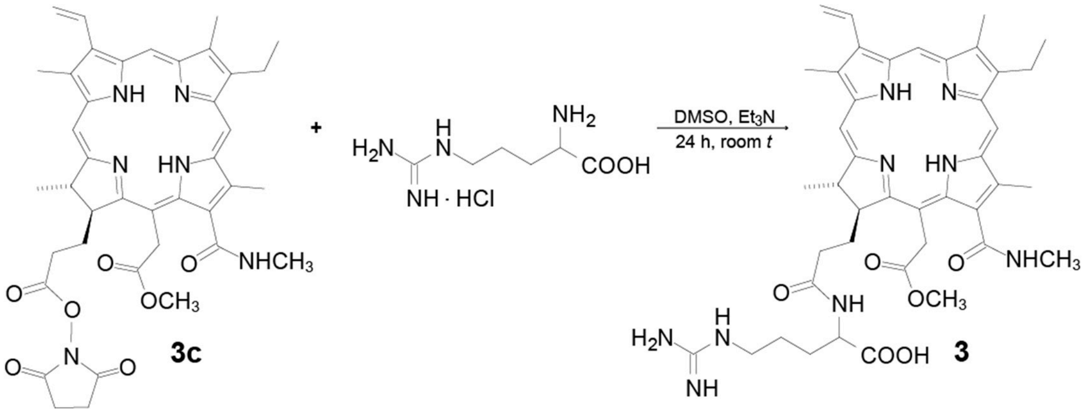

Scheme S5: Synthesis of chlorin e6 13(1)-N-methylamide-15(2)-methyl ester-17(3)-[N-1′-(1′-carboxy-4′-guanidylbutyl) amide] (comp. **3**).

158.6 mg (0.22 mmol) of chlorin e6 13(1)-N-methylamide-15(2)-methyl ester-17(3)-N-succinimide ester (**3c**), 46.4 mg (0.22 mmol) of arginine hydrochloride, 10 ml of dimethylsulfoxide (DMSO) and 0.5 ml of trimethylamine (TMA) were mixed (equivalent amount of diisopropylethylamine can be used instead TMA). The reaction mixture was stirred for 24 hours at a room temperature. Then 10 ml of chloroform was added to the chlorin solution, the organic layer was washed with water containing a few drops of acetic acid and, finally, with pure water twice. The organic layer was separated and the solvent was evaporated. The resulting crude product was chromatographed on the column filled with a silica gel using the dichloromethane-methanol mixtures as an eluent. The product yield is about 30 mg (total 17%).

The MS and NMR spectra for comp. **3** are presented in Figures S8-S10.

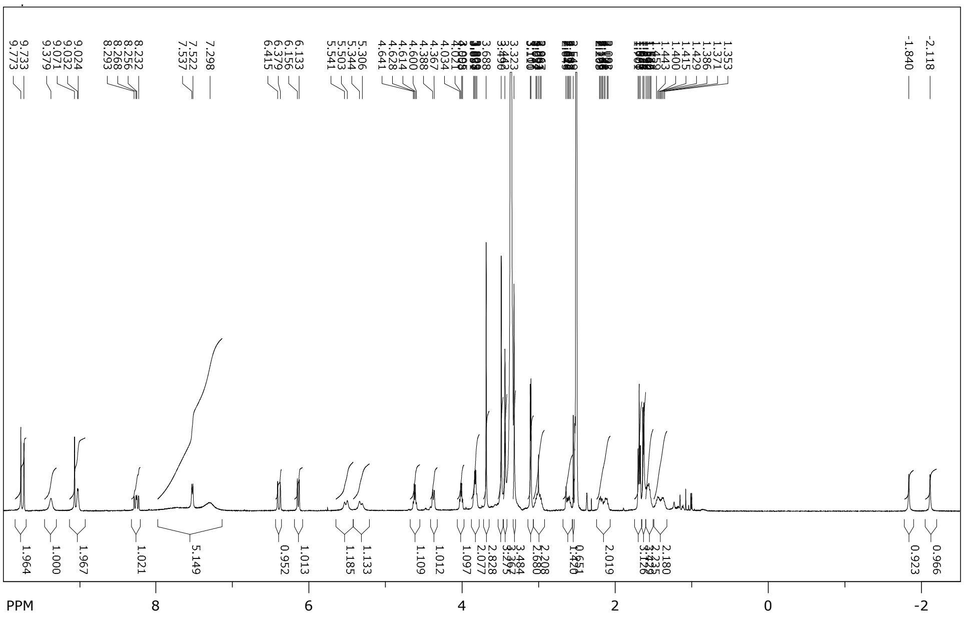

Figure S8: ^{1}H NMR spectrum of chlorin e6 13(1)-N-methylamide-15(2)-methyl ester-17(3)-[N-1′-(1′-carboxy-4′-guanidylbutyl) amide] (comp. **3**) in $CDCl_3$.

Mass spectrum m/z (MALDI) (%): M = 781.5 (100) $[M]^+$; 803.5 (28) $[M+Na]^+$; 825.5 (5) $[M+K]^+$. Calculated: $C_{42}H_{54}N_9O_6$, M = 780.9349.

^{1}H NMR spectrum (500 MHz, DMSO d_6), δ, ppm: 9.77 (s, 1H, 10-H); 9.73 (s, 1H, 5-H); 9.38 (br. s., 1H, 17(3)-NH); 9.07 (s, 1H, 20-H); 9.03 (d, *J* = 4.0 Hz, 1H, 13-NCH_3); 8.26 (dd, *J* = 11.8, 17.9 Hz, 1H, 3(1)-H); 6.85-8.05 (br. m., 5H, NH, guanidine group); 6.40 (d, *J* = 17.8 Hz, 1H, 3(2)-H-trans); 6.15 (d, *J* = 11.7 Hz, 1H, 3(2)-H-cis); 5.52 (d, *J* = 18.8 Hz, 1H, 15(1)-CH); 5.32 (d, *J* = 18.5 Hz, 1H, 15(1)-CH); 4.62 (dd, *J* = 6.9, 14.2 Hz, 1 H, 18-H); 4.38 (d, *J* = 10.35 Hz, 1H, 17-H); 4.02 (dd, *J* = 6.4, 13.2 Hz, 1H, C1'-CH); 3.83 (q, *J* = 6.8 Hz, 2H, 8(1)-CH_2); 3.68 (s, 3H, 15(3)-CH_3); 3.49 (s, 3H, 12(1)-CH_3); 3.44 (s, 3H, 2(1)-CH_3); 3.32 (s, 3H, 7(1)-CH_3); 3.11 (d, *J* = 4.4 Hz, 3H, 13(1)-NCH_3); 3.00 (m, 2 H, C4'-CH_2); 2.55 (s, 1H); 17(1)-CH_2, 17(2)-CH_2): 2.12, 2.19 (2m, 1H each), 2.57-2.66 (m, 2H); 1.68 (t, *J* = 7.5 Hz, 3H, 8(2)-CH_3); 1.63 (d, *J* = 7.2 Hz, 3H, 18(1)-CH_3); 1.56 (m, 2H, C2'-CH_2); 1.38, 1.44 (2m, 1H each, C3'-CH_2); -1.84 (s, 1H, 21-NH); -2.12 (s, 1H, 23-NH).

^{13}C NMR spectrum (DMSO d_6, 500 MHz), δ, ppm: 179.81 ($COOH^{17(6)}$), 172.96 ($COO^{15(2)}CH3$), 169.59 ($CO^{13(1)}NHCH3$), 168.71 ($CO^{17(3)}$ NH-Arg), 157.18 ($C^{17(11)}$=NH-Arg), 152.82, 148.42, 144.30, 137.75 (C^{4}), 136.19 (C^{2}), 135.04 (C^{12}), 134.72, 133.60, 130.02 (C^{3}), 129.87, 129.38 ($CH^{3(1)}$), 121.86 ($CH_2{}^{3(2)}$), 102.76, 100.66 (CH^{10}), 98.50 (CH^{5}), 94.15 (CH^{20}), 92.50, 73.55, 63.08, 53.52 (CH^{17}), 52.87 ($CH_3{}^{15(3)}$) , 51.80 ($CH_2{}^{8(1)}$), 48.19, 40.62 ($CH_3{}^{13(3)}$), 36.63, 32.61 and 30.73 ($CH_2{}^{17(1)}$ and $CH_2{}^{17(2)}$), 29.86 and 25.44 ($CH^{17(5)}$, $CH_2{}^{17(7)}$, $CH_2{}^{17(8)}$, $CH_2{}^{17(9)}$), 26.59 ($CH_3{}^{7(1)}$), 23.04 ($CH_3{}^{8(2)}$), 18.95 (CH^{18}), 17.87 ($CH_3{}^{18(1)}$), 12.03 ($CH_3{}^{12(1)}$), 11.72 ($CH_3{}^{2(1)}$), 11.04.

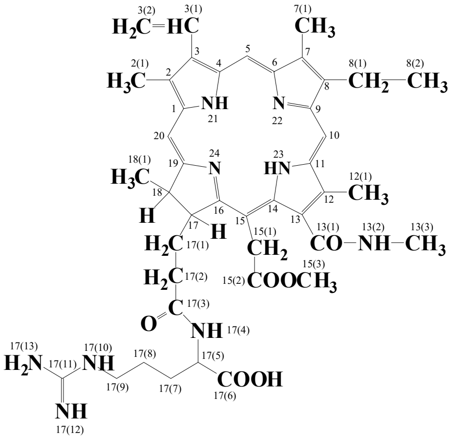

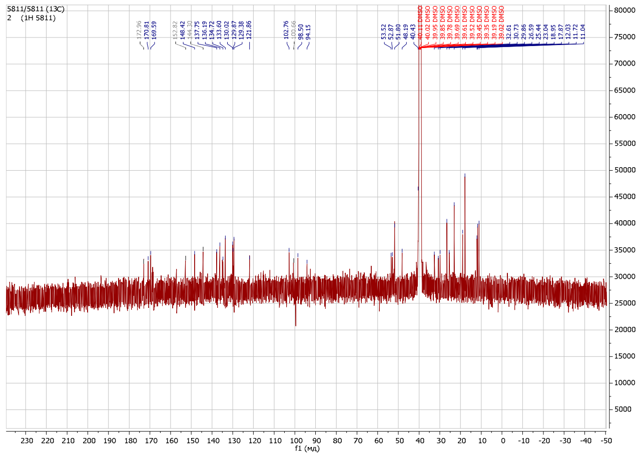

Figure S9: ^{13}C NMR spectrum of chlorin e_6 13(1)-N-methylamide-15(2)-methyl ester-17(3)-[N-1′-(1′-carboxy-4′- guanidylbutyl) amide] (comp. **3**) in DMSO d_6.

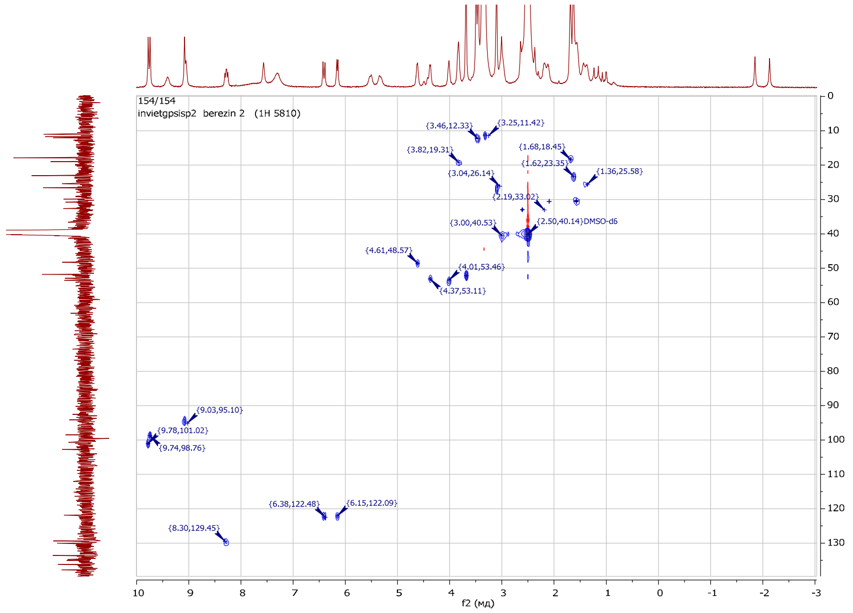

Figure S10: General view of the two-dimensional ^{1}H - ^{13}C NMR spectrum (HSQC) of chlorin e_6 13(1)-N-methylamide-15(2)-methyl ester-17(3)-[N-1′-(1′-carboxy-4′-guanidylbutyl) amide] (comp. **3**) in DMSO d_6 (m = 5 10^{-3} mol/kg).

1.4 Chlorin e_6 3(1),3(2)-*bis*-(N,N,N-trimethylaminomethyl iodide)-13(1)-N'-(2-N'',N'',N''-trimethyl ammonioethyl iodide) amide 15(2),17(3)-dimethyl ester (comp. 4)

The tricationic chlorin (comp. **4**) was obtained by chemical functionalization of methylpheophorbide *a* described earlier [S2] (see Scheme S6).

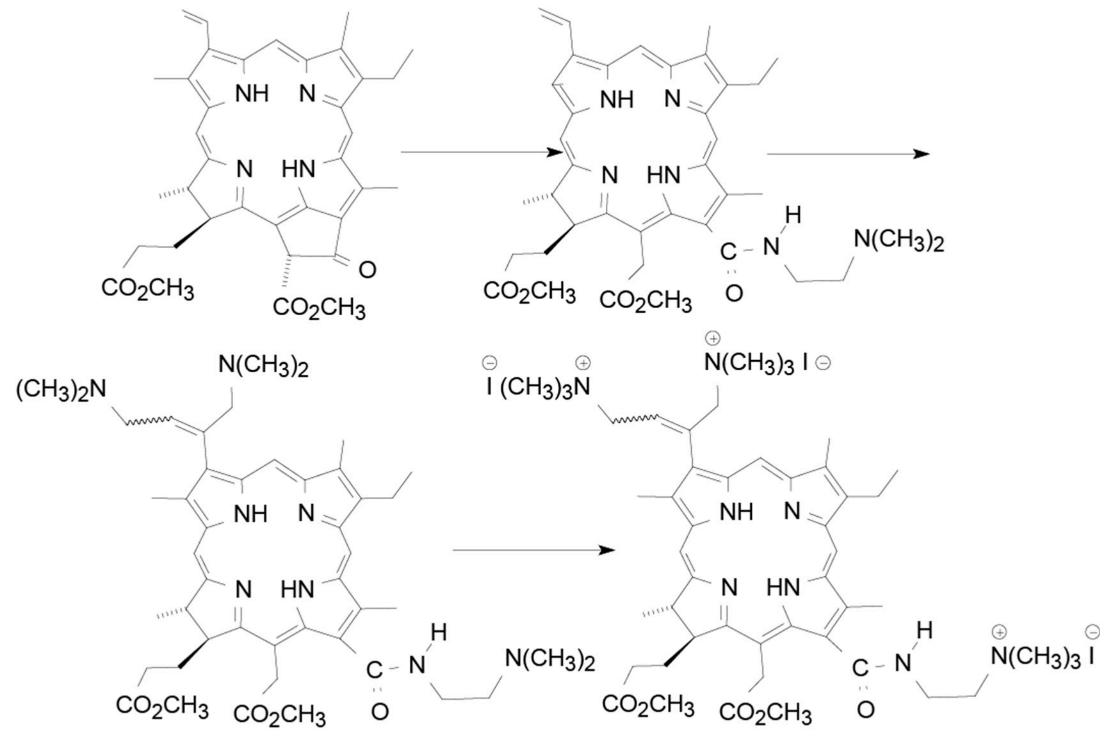

4

Scheme S6: Synthesis of chlorin e_6 3(1),3(2)-*bis*-(N,N,N-trimethylaminomethyl iodide)-13(1)-N'-(2-N'',N'',N''-trimethyl ammonioethyl iodide) amide 15(2),17(3)-dimethyl ester (comp. **4**).

The NMR spectra for comp. **4** are presented in Figure S11.

MS (ESI) m/z ($M^{3+}I_3$) (MI_3): 968.8 ($([M^{3+}+2H-CH_2{}^{+}]I)^{+}$), ($([MI_3+2H-CH_3I-I^{-}))^{+}$), 923.3 ($([M^{3+}+2H-N(CH_3)_3{}^{+}]I)^{+}$), 421.1 ($(M^{3+}+4H-CH_2{}^{+})^{2+}$ or $(MI_3+4H-CH_3I-2I^{-})^{2+}$. MS (MALDI-TOF-MS, CHCA as matrix): Found $[M-3HI+Sr]^{+}$ 938.5858 and $[M-3I-CH_2N(CH_3)_3-2(CH_3)_3N]^{+}$ 662.3758; molecular formula $C_{49}H_{73}N_8O_5I_2$ requires $[M-3HI+Sr]^{+}$ 938.5700 and $[M-3I-CH_2N(CH_3)_3-2(CH_3)_3N]^{+}$ 662.3110.

^{1}H NMR (DMSO d_6), δ, ppm: 9.87 (2 H, s, 10-H, 5-H); 8.59-8.46 (br.m, 1H, 13-$CON\underline{H}CH_2CH_2N^{+}(Me)_3I^{-}$); 9.17 (s, 1H, 20-H); 7.39-7.22 (br.m, 1H, 3-$C(CH_2N^{+}(Me)_3I^{-})=C\underline{H}CH_2N^{+}(Me)_3I^{-}$); 5.50 (d, J = 19.2 Hz, 1H, 15-$C\underline{H_A}H_BCO_2Me$); 5.35 (d, J = 19.2 Hz, 1H, 15-$CH_A\underline{H_B}CO_2Me$); 4.67 (q, J = 7.3 Hz, 1H, 18-H); 4.48 (br.d, J = 8.3 Hz, 1H, 17-H); 4.13-3.99 (m, 2H, 13-$CONHC\underline{H_2}CH_2N(Me)_2$); 3.96-3.82 (m, 2H, 8-$C\underline{H_2}Me$); 3.88 (s, 3H, 15-$CH_2CO_2Me$); 3.76 (s, 3H, 17-$CH_2CH_2CO_2\underline{Me}$); 3.59 (s, 6H, 12-Me, 7-CH_3); 3.57 (s, 3H, 2-Me); 3.39 (s, 18H, 3-$C(CH_2N^{+}(\underline{Me})_3I^{-})=CHCH_2N^{+}(\underline{Me})_3I^{-})$); 3.16 (s, 9H, 13-$CONHCH_2CH_2N^{+}(\underline{Me})_3$); 3-$C(C\underline{H_2}N^{+}(Me)_3I^{-})=CHC\underline{H_2}N^{+}(Me)_3I^{-}$: 3.05-2.88 (m, 2H), 2.83-2.65 (2H, m); 2.34-2.24 (m, 2H, 13-$CONHCH_2C\underline{H_2}N^{+}(Me)_3I^{-}$), 2.21-1.89 (m, 4H, 17-$C\underline{H_2}C\underline{H_2}CO_2Me$), 1.72 (t, 3H, J = 7.3 Hz, 8-CH_2Me), 1.67 (d, J = 7.2 Hz, 3H, 18-Me), -1.82 (br.s, 1H, 23-NH), -2.09 (br.s, 1H, 21-NH).

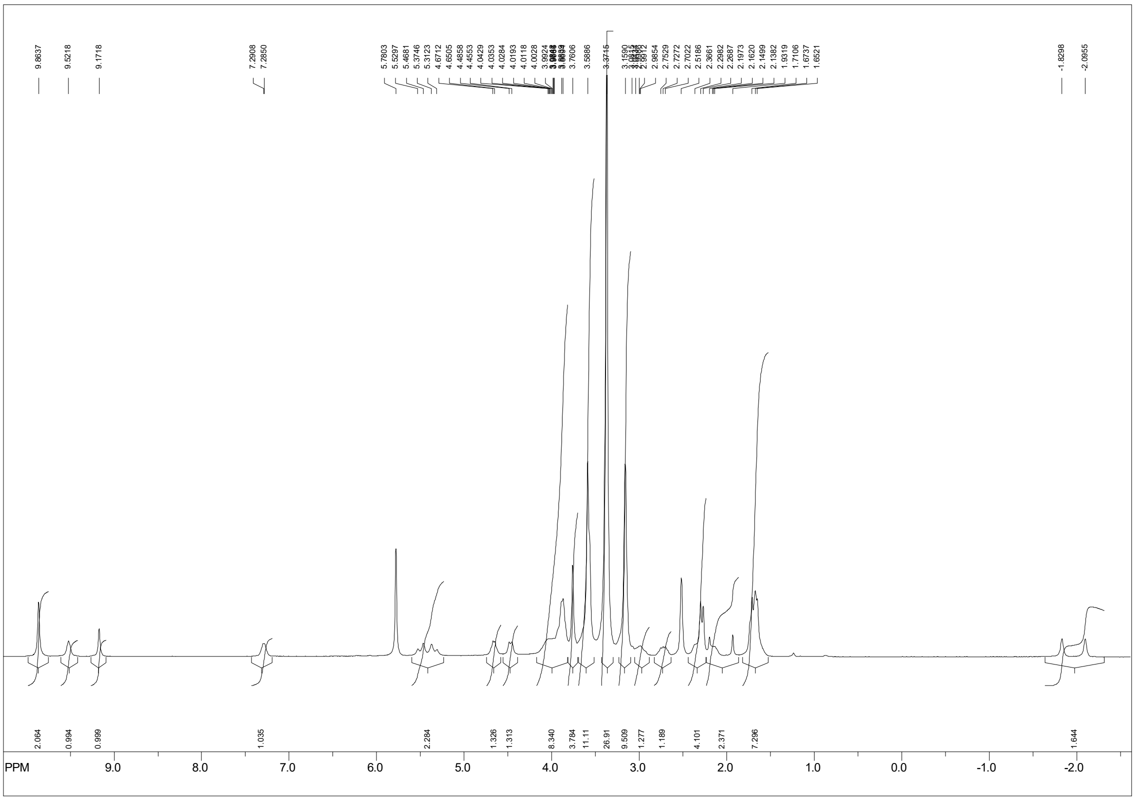

Figure S11: ^{1}H NMR spectrum of chlorin e_6 3(1),3(2)-*bis*-(N,N,N-trimethylaminomethyl iodide)-13(1)-N'-(2-N'',N'',N''-trimethylammonioethyl iodide) amide 15(2),17(3)-dimethyl ester (comp. **4**) in DMSO d_6

2. Spectroscopic measurements

The results of spectrophotometric and fluorescence titration of comps. **1**-**4** by Tween 80 and potassium iodide are given in Tables S1 and S2.

Table S1. Titration of comps. **1**-**4** in aqueous solutions with surfactant Tween 80

Comp. **1**, λ= 670 nm		Comp. **2**, λ= 669 nm		Comp. **3**, λ= 670 nm		Comp. 4, λ=395 nm	
$m_{\text{Tween 80}}\cdot10^4$, mol/kg	*A*, r.u.	$m_{\text{Tween 80}}\cdot10^4$, mol/kg	*A*, r.u.	$m_{\text{Tween 80}}\cdot10^4$, mol/kg	*A*, r.u.	$m_{\text{Tween 80}}\cdot10^4$, mol/kg	*A*, r.u.
0	0.028	0	0.074	0	0.069	0	1.290
0.229	0.060	0.347	0.164	4.33	0.095	0.062	1.332
0.442	0.078	0.682	0.185	6.37	0.101	0.113	1.346
0.667	0.092	0.940	0.193	8.05	0.115	0.145	1.349
0.88	0.100	1.82	0.200	10.8	0.122	0.237	1.366
1.08	0.106	3.05	0.211	11.3	0.130	0.261	1.375
1.30	0.114	4.21	0.221	14.1	0.132	0.533	1.387
1.53	0.120	5.60	0.231	16.7	0.136	0.557	1.39
1.76	0.128	7.16	0.240	20.3	0.153	0.874	1.438
2.78	0.135	8.08	0.246	24.2	0.162	1.11	1.441
3.63	0.140	8.72	0.250	25.4	0.163	1.48	1.479
5.44	0.141	11.7	0.254	26.5	0.164	2.21	1.480
6.27	0.142	13.9	0.256	28.2	0.165	2.55	1.481

Table S2. Fluorescence titration of aqueous Tween 80 solutions of comps. **1-4** by KI at various surfactant/ molar ratios

Comp. **1**		Comp. **2**		Comp. **3**		Comp. **4**	
m_{KI}, mol/kg	F/F_0	m_{KI}, mol/kg	F/F_0	m_{KI}, mol/kg	F/F_0	m_{KI}, mol/kg	F/F_0
			Tween 80/PS ratio =60				
0	1					0	1
0.010	1.039					0.073	1.695
0.022	1.105					0.142	1.922
0.032	1.155					0.209	2.471
0.060	1.235					0.272	2.915
0.085	1.324					0.333	3.270
0.122	1.405					0.446	3.783
0.173	1.515					0.551	4.173
0.397	1.940					0.647	4.450
0.468	2.171					0.735	5.068
0.542	2.342					0.818	5.218
0.615	2.604					0.966	5.655
0.760	3.059						
0.850	3.271						
1.02	3.853						
1.16	4.121						
1.32	4.524						
1.56	5.079						
			Tween 80/PS ratio = 200				
0	1	0	1	0	1	0	1
0.016	1.020	0.025	1.064	0.023	1.088	0.024	1.141
0.033	1.029	0.075	1.075	0.036	1.131	0.057	1.318
0.050	1.048	0.088	1.078	0.057	1.144	0.091	1.471
0.087	1.085	0.107	1.149	0.141	1.262	0.133	1.664
0.171	1.143	0.140	1.152	0.182	1.309	0.184	1.836
0.299	1.229	0.175	1.144	0.204	1.348	0.234	2.004
0.439	1.322	0.211	1.164	0.245	1.360	0.323	2.236
0.587	1.429	0.277	1.215	0.336	1.509	0.461	2.490
0.750	1.540	0.353	1.262	0.406	1.534	0.588	2.790
0.922	1.653	0.435	1.328	0.504	1.633	0.736	3.001
1.07	1.771	0.539	1.400	0.598	1.707	0.840	3.221
		0.647	1.405	0.691	1.736	0.937	3.396
		0.798	1.535	0.770	1.852	1.04	3.530
		0.935	1.560	0.849	1.845	1.17	3.682
		1.03	1.648	0.930	1.909	1.31	3.836
		1.12	1.635	1.04	1.978	1.51	4.127
		1.22	1.639	1.15	2.071		
		1.39	1.597	1.54	2.243		
		1.64	1.705				

References:

[S1] Kustov, A.V.; Privalov, O.A.; Strelnikov, A.I.; Koifman, O.I.; Lubimtsev, A.V.; Morshnev, Ph.K.; Moryganova, T.M.; Kustova, T.V.; Berezin, D.B. Transurethral resection of non-muscle invasive bladder tumors combined with fluorescence diagnosis and photodynamic therapy with chlorin e_6-type photosensitizers. *J. Clin. Med.* **2022**, *11*, 233. DOI: 10.3390/jcm11010233

[S2] Kustov, A.V.; Belykh, D.V.; Smirnova, N.L.; Venediktov, E.A.; Kudayarova, T.V.; Kruchin, S.O.; Khudyaeva, I.S.; Berezin, D.B. Synthesis and investigation of water-soluble chlorophyll pigments for antimicrobial photodynamic therapy. *Dyes Pigm.* **2018**, *149*, 553-559. DOI:10.1016/j.dyepig.2017.09.073

[S3] Mal'shakova, M.V.; Belykh, D.V.; Velegzhaninov, I.O.; Rasova, E.E. Novel chlorophyll *a* derivatives with ester-linked galactose fragments for photodynamic therapy and fluorescence diagnostics of cancer. *J. Porph. Phthaloc.* **2020**, *25*, 135-144. DOI: 10.1142/S1088424621500048

[S4] Belykh, D.V. Synthesis of polyfunctional chlorins and its analogues based on methylpheophorbide *a*. ICKSC UB RAS Edit: Syktyvkar, Russia, 2012. 162 p. (in Russian).
